# Rapid and transient enhancement of thalamic information transmission induced by vagus nerve stimulation

**DOI:** 10.1088/1741-2552/ab6b84

**Published:** 2020-04-08

**Authors:** Charles Rodenkirch, Qi Wang

**Affiliations:** Department of Biomedical Engineering, Columbia University, ET351, 500 W. 120th Street, New York, NY 10027, United States of America

**Keywords:** vagus nerve stimulation (VNS), sensory processing, thalamus, feature selectivity, fast duty cycle

## Abstract

**Objective.:**

Vagus nerve stimulation (VNS) has been FDA-approved as a long-term, therapeutic treatment for multiple disorders, including pharmacoresistant epilepsy and depression. Here we elucidate the short-term effects of VNS on sensory processing.

**Approach.:**

We employed an information theoretic approach to examine the effects of VNS on thalamocortical transmission of sensory-related information along the somatosensory pathway.

**Main results.:**

We found that VNS enhanced the selectivity of the response of thalamic neurons to specific kinetic features in the stimuli, resulting in a significant increase in the efficiency and rate of stimulus-related information conveyed by thalamic spikes. VNS-induced improvements in thalamic sensory processing coincided with a decrease in thalamic burst firing. Importantly, we found VNS-induced enhancement of sensory processing had a rapid onset and offset, completely disappearing one minute after cessation of VNS. The timescales of these effects indicate against an underlying mechanism involving long-term neuroplasticity. We found several patterns of VNS (tonic, standard duty-cycle, and fast duty-cycle) all induced similar improvements in sensory processing. Under closer inspection we noticed that due to the fast timescale of VNS effects on sensory processing, standard duty-cycle VNS induced a fluctuating sensory processing state which may be sub-optimal for perceptual behavior. Fast duty-cycle VNS and continuous, tonic VNS induced quantitatively similar improvements in thalamic information transmission as standard duty-cycle VNS without inducing a fluctuating thalamic state. Further, we found the strength of VNS-induced improvements in sensory processing increased monotonically with amplitude and frequency of VNS.

**Significance.:**

These results demonstrate, for the first time, the feasibility of utilizing specific patterns of VNS to rapidly improve sensory processing and confirm fast duty-cycle and tonic patterns as optimal for this purpose, while showing standard duty-cycle VNS causes non-optimal fluctuations in thalamic state.

## Introduction

The vagus nerve, the longest cranial nerve and part of the parasympathetic nervous system, originates from the medulla and innervates organs in the thorax and abdomen. The majority of vagus nerve afferent fibers project to the brain through the nucleus tractus solitarius (NTS) (George et al 2000, Groves and Brown 2005). However, in addition to this pathway the vagus nerve also has ipsilateral projections to the area postrema, dorsal motor nucleus of the vagus, nucleus ambiguus, medullary reticular formation, and the spinal trigeminal nucleus (Krahl and Clark 2012). Vagus nerve stimulation (VNS) has long been known to have profound effects on the neural dynamics of the central nervous system. Due to the ease at which the vagus nerve can be accessed, VNS has attracted tremendous interest from the clinical community and has been FDA-approved for many different treatments, including therapies for intractable epilepsy and pharmacoresistant depression (Pisapia and Baltuch 2015, Yuan and Silberstein 2016, Gonzalez et al 2019). In the last decade, numerous efforts have been made to test the efficacy of VNS in treating a wide variety of other neurological and psychiatric disorders, including autism, stroke-induced damage, PTSD, pain, inflammation, addiction, and obesity (Engineer et al 2011, Khodaparast et al 2014, Chakravarthy et al 2015, Childs et al 2017, Lamb et al 2017, Romero-Ugalde et al 2017, Conway et al 2018, Pelot and Grill 2018, Huffman et al 2019, Spindler et al 2019, van Hoorn et al 2019). More recently transdermal VNS has been suggested as a potential method of non-invasively activating the vagus nerve (Mourdoukoutas et al 2018, Mwamburi et al 2017, Hamer and Bauer 2019, Reuter et al 2019), as evidenced by the FDA approving commercial non-invasive VNS systems for use in clinical applications.

The vagus nerve presumably modulates brain circuit dynamics through influence on several neuro-modulatory systems, including the locus coeruleus—norepinephrine (LC-NE) system (Dorr and Debonnel 2006). Elevated firing rate of LC neurons in response to VNS has been confirmed by several previous studies (Groves et al 2005, Hulsey et al 2017). Indeed, previous work has suggested that the LC is one of the main brain structures mediating the beneficial therapeutic effects of VNS on abnormal brain activity. For example, the ability of VNS to abort seizures was significantly reduced after lesioning of the LC (Krahl et al 1998). The LC is the primary source of NE to the forebrain and as such plays a pivotal role in many brain functions. Our recent work demonstrated that LC activation dramatically improved information transmission in the somatosensory thalamus, a critical stage for sensory processing and perceptual performance. Moreover, we found the mechanism underlying this improvement was LC-NE mediated suppression of burst firing in the intra-thalamic circuitry via direct action of NE on thalamic noradrenergic receptors. Reduction of calcium t-channel activity, the membrane channels responsible for burst firing, led to an increased ability of thalamic relay neurons to respond selectively to the specific kinetic features each neuron encodes (Roden-kirch et al 2019). We therefore reasoned it may be possible to utilize VNS as a method for enhancement of sensory processing. However, the extent to which VNS affects thalamic information transmission had not been examined.

In this study, we sought to investigate the effects of VNS on the feature selectivity and information transmission of neurons of the ventroposterior medial nucleus of thalamus (VPm), the thalamic relay stage of the rat vibrissa system. We recorded single-unit VPm responses to white Gaussian noise (WGN) whisker stimulation while systematically varying VNS patterns. We found VNS significantly enhanced the feature selectivity of VPm neurons, resulting in improved transmission of sensory-related information. Interestingly, this improvement was similar to that induced by direct LC stimulation as both VNS and direct LC stimulation reduced VPm burst firing. Previous VNS techniques have focused on facilitating long-term, neuroplastic change of brain circuits (Hays et al 2013, Buell et al 2019). In contrast, here we found VNS was also able to produce rapid and short-lasting effects on thalamic feature selectivity and information transmission, evidenced by the fact that thalamic information transmission returned to baseline conditions approximately 60 s after VNS cessation.

In clinical applications, VNS is commonly delivered in a duty-cycle fashion, with a standard pattern consisting of on periods consisting of 30 s of continuous VNS delivered at 30 Hz interleaved with off periods of 60 s or longer (Heck et al 2002, DeGiorgio et al 2005, Yamamoto 2015, Musselman et al 2018). Having found that VNS-induced enhancement of sensory processing rapidly dissipated following cessation of VNS, we wondered how duty-cycle VNS with standard off periods would affect the sensory processing state of the thalamus. As expected, we found that improvement in information transmission was significantly stronger during the 30 s VNS on period when compared to the second 30 s of the VNS off period of standard duty-cycle VNS. This indicates that standard duty-cycle VNS creates a fluctuating thalamic sensory processing state. Such a state would be detrimental for discrimination of stimuli, as the same stimulus would evoke different VPm responses depending on if it was received during the on or off period of the standard duty-cycle VNS.

To find a VNS pattern that could be safely used to enhance sensory processing without inducing a fluctuating state we also examined VNS with a fast duty-cycle (i.e. 3 s on 7 s off) and 10 Hz tonic VNS. We found these patterns both induced quantitatively similar improvement in thalamic information transmission when compared to standard duty-cycle VNS. Importantly we found that fast duty-cycle VNS did not produce fluctuations in sensory processing as evidenced by equal rates of burst spiking and information improvement found to occur during the on period versus the second half of the off period. Further, both fast duty-cycle and tonic VNS-induced improvements increased monotonically with increased VNS amplitude and tonic VNS-induced improvements increased monotonically with increased VNS frequency. Taken together, our results have demonstrated, for the first time, that VNS is able to rapidly enhance information processing in the sensory system. Moreover, our data suggested that specific patterns of VNS without long off periods, such as fast duty-cycle and tonic VNS, should be used for VNS-enhancement of information transmission as the relatively long off periods used by standard dutycycle VNS create a non-optimal fluctuating sensory processing state.

## Material and methods

All animal work was approved by the Columbia University Institutional Animal Care and Use Committee and the procedures were conducted in compliance with NIH guidelines. 16 adult albino rats (Sprague-Dawley, Charles River Laboratories, Wilmington, MA; ~225–275 g at time of implantation) were used in this study. Animals were housed 1–2 per cage in a dedicated housing facility, which maintained a twelve-hour light and dark cycle.

### Surgery

Rats were sedated with 5% vaporized isoflurane in their home cages before being transported to the surgery suite at 2% vaporized isoflurane. Rats were then mounted on a stereotaxic frame, and the anesthetic was switched to ketamine/xylazine (80/8 mg kg^−1^) (Hulsey et al 2017). Body temperature was kept at 37 °C by a servo-controlled heating pad (FHC Inc, Bowdoin, ME). Blood-oxygen saturation level and heart rate were continuously monitored using a non-invasive monitor (Nonin Medical Inc., Plymouth, MN).

To allow for implantation of the VNS cuff, an incision was made on the left ventral side of the body. A magnetic fixator retraction system (Fine Scientific Tools, Foster City, CA) was used to separate the sternohyoid and sternomastoid muscles longitudinally, providing clear access to the vagus nerve running next to the carotid artery within the carotid sheath. Glass tools were used to separate the vagus nerve from the carotid sheath to minimize any potential damage to the nerve. A platinum–iridium bipolar cuff electrode (Rios et al 2019) was then placed around the vagus nerve to allow for delivery of VNS. An insulated lead connected to the VNS cuff was then ran out of the incision, which was closed with sutures.

Following VNS implantation, the animal was carefully mounted on a custom-modified stereotaxic frame (RWD Life Science, China) on top of a floating air table so that a craniotomy could be created above the VPm to allow for insertion of a recording electrode. On a subset of animals which did not have a VNS cuff implanted, a second craniotomy was also opened above the LC to allow for direct LC stimulation (3 rats). Retaining wells were created around the craniotomies to allow for any exposed brain surface to be covered in warm saline.

### Electrophysiology

Single, sharp, tungsten microelectrodes (75 *μ*m in diameter, impedance of ~3–5 MΩ, FHC Inc, Bowdoin, ME) were used to record extracellular single-unit activity. A hydraulic micropositioner (David Kopf, Tujunga, CA) allowed for slow, controlled electrode positioning with micrometer resolution, and thus allowed for close proximity placement to recorded neurons. Extracellular neural signals were referenced to a ground screw in contact with the surface of the dura, contralateral to the recording site, then bandpass filtered (300–8k Hz) and digitized at 40 kHz using a Plexon recording system (OmniPlex, Plexon Inc., Dallas, TX). Spike sorting of single units was performed using commercially available software (Offline Sorter, Plexon).

The VPm was targeted using stereotaxic coordinates from the rat brain atlas (Paxinos and Watson 1998). VPm neuron identity was confirmed by a strong response to the mechanical stimulation of the neuron’s principal whisker (Wang et al 2010, 2012, Millard et al 2013). Only large, easily isolatable VPm units with a minimum refractory period greater than 1 ms and a stable waveform throughout the entire recording were used. Burst spiking was defined as any two or more spikes occurring with an ISIs of 4 ms or less and following at least 100 ms of quiescence (Sherman 2001).

To estimate the effect of direct LC stimulation on thalamic sensory processing, in some experiments, a tungsten microelectrode with a lower impedance (~2 MΩ, FHC Inc., Bowdoin, ME) was used to first electrophysiologically locate and subsequently microstimulate the LC. LC neuron identity was confirmed by a wide spike waveform and biphasic response to a paw pinch (Liu et al 2017, Rodenkirch et al 2019).

### Vagus nerve stimulation (VNS)

The vagus nerve cuff lead was connected to a calibrated electrical microstimulator (Multi Channel Systems, Reutlingen, Germany) which was triggered by an xPC target real-time system (MathWorks, MA) running at 1 kHz. During periods of VNS, cathodeleading biphasic current pulses (250 *µ*s per phase) were delivered at either 10 or 30 Hz with amplitudes of either 0.4, 1, or 1.6 mA with duty-cycles of either continuous, fast (3 s on/7 s off), or standard (30 s on/60 s off). For each recording, multiple repetitions of each VNS condition were delivered in a random order. Each VNS condition delivery lasted 180 s with 75–90 s of rest time inserted following to allow for the system to reset to baseline conditions before beginning the next condition. As currently practiced in humans, only the left vagus nerve was stimulated as stimulation of the right vagus nerve has been shown to cause cardiac irregularities due to right vagus nerve efferents innervating the sinoatrial node (Ben-Menachem 2001). Further, the polarity of VNS was fixed (negative electrode cranial) as a reversal of this polarity has been shown to induce bradycardia (Asconape et al 1999).

### LC microstimulation

Microstimulation of the LC was described in detail previously (Liu et al 2017, Rodenkirch et al 2019). Briefly, after the LC was electrophysiologically confirmed, the recording microelectrode was disconnected from the recording system and connected to a calibrated electrical microstimulator (Multi Channel Systems, Reutlingen, Germany), which was then triggered by an xPC target real-time system running at 1 kHz. During periods of microstimulation of the LC, cathode-leading biphasic current pulses (200 *µ*s/phase, 60 *µ*A) were continuously delivered at either 2 or 5 Hz. Each LC activation condition was delivered, in a random order, beginning 5 s before whisker stimulation and lasting throughout the entire period of whisker stimulation for a total length of 165 s. Ninety-five seconds of dead time was inserted between each stimulation period to allow for the system to return to baseline conditions.

### Whisker stimulation

A custom modified galvo motor (galvanometer optical scanner model 6210H, Cambridge Technologies) controlled by a closed-loop system (micromax 67 145 board, Cambridge Technology) as described in Chagas et al (2013) was used to deliver precise, high-frequency mechanical whisker stimulations (12.5 mm shaft). The galvo motor’s position was controlled via the same xPC target real-time system controlling VNS/LC activation. Accuracy of whisker stimulation was verified by using the Plexon recording system to also record the galvo motor’s output analog position signal. Whiskers were cut to a length of ~10 mm and inserted into the deflecting arm, which was positioned ~5 mm from whiskerpad. The WGN was low pass filtered (butterworth, 10th order) at 250 Hz (Rodenkirch et al 2019). The galvo motor was used to continuously deliver whisker deflection following a signal consisting of continuous repetitions of a 15 s clip of frozen white Gaussian noise (WGN). As we aimed to determine if neurons had similar or altered responses to identical stimuli under varying conditions of VNS, the plane of whisker deflection was fixed throughout the recording.

### Data analysis

Here, we assume VPm neurons encode for stimulus-related information via the linear-nonlinear-Poisson model (LNP) as previously detailed by Schwartz et al (2006), Petersen et al (2008) and Rodenkirch et al (2019). Through analyzing the neuron’s spiking response to a repeated delivery of a frozen WGN whisker deflection pattern, we can recover the neuron’s feature selectivity, which can be represented by a linear filter set and the corresponding set of nonlinear tuning functions. Specifically, each neuron’s first significant feature was recovered as the spike triggered average (STA) whisker displacement during the 20 ms window preceding each spike. Spike triggered covariance (STC) analysis was then used to recover the remaining set of significant features for any neurons which selectively responded to more than one kinetic feature (Schwartz et al 2006).


$$\;\text{STA}=\frac{1}{N}\sum_{n=1}^{N}\vec{S}\left(t_{n}\right)\;\text{STC}=\frac{1}{N-1}\sum_{n=1}^{N}\left[\vec{S}\left(t_{n}\right)-\text{STA}\right]{\left[\vec{S}\left(t_{n}\right)-\text{STA}\right]}^{T}.$$

Where *t_n_* is the time of the *n*th spike, *S* (*t_n_*) is a vector representing the stimulus during the temporal window preceding a spike, and N is the total number of spikes.

Statistical significance of STAs was determined using a bootstrap procedure with 1000 bootstrap trials. Recovered STAs were considered insignificant if their amplitude fell within the 99.9 percentile of the bootstrap displacement range. The significance of STC recovered filters was determined using nestled bootstrapping of the eigenvalues corresponding to the STC recovered filters. A recovered eigenvalue that exceeded the 99.9 percentile of its corresponding bootstrap range of its filter was considered significant. Neurons without significant feature selectivity across all stimulation conditions were excluded from further analysis.

To quantify the modulation of the recovered features by LC activation, we defined a feature modulation factor as (Rodenkirch et al 2019):

$$\text{feature}\;\text{modulation}\;\text{factor}=\frac{\text{control}\;\text{feature}\;\cdot \text{conditional}\;\text{feature}}{\text{control}\;\text{feature}\;\cdot \text{control}\;\text{feature}}.$$

To estimate each nonlinear tuning function corresponding to each significant recovered feature, we first calculated the feature coefficient for each spike, i.e. the dot product between a neuron’s linear filter and the stimulus preceding each spike. The probability distribution of feature coefficient values k given a spike (i.e. Prob(k|spike)) could then be determined. To calculate all possible feature coefficients for the stimulus used, a 20 ms window was slid through the 15 s WGN stimulus, from which a probability distribution of all feature coefficient values (i.e. Prob(k)) was generated. By dividing Prob(k|spike) by Prob(k), we produced the nonlinear tuning functions that map firing rate to feature coefficient value.

To quantify the information the spike train conveys about the absence/presence of a feature under varying VNS or LC stimulation conditions, we calculated mutual information between the presence/absence of a feature and the observation of a spike for each condition as (Adelman et al 2003)

$$\text{Info}\left(k;\text{spike}\right)=\int dk*\text{Prob}\left(k|\text{spike}\right)*\log_{2}\left(\frac{\text{Prob}\left(k|\text{spike}\right)}{\text{Prob}\left(k\right)}\right).$$

Where *k* is the feature. Information transmission rate (i.e. bits/second) was calculated by multiplying bits/spike by the average firing rate of the neuron in response to WGN stimulus.

### Statistics

A one-sample Kolmogorov-Smirnov test was used to assess the normality of data before performing statistical tests. If the samples were normally distributed, a paired or unpaired *t*-test was used. Otherwise, the Mann–Whitney *U*-test was used for unpaired samples or the Wilcoxon signed-rank test for paired samples. Multiple comparisons were corrected with Bonferroni correction.

## Results

**Figure 1.**
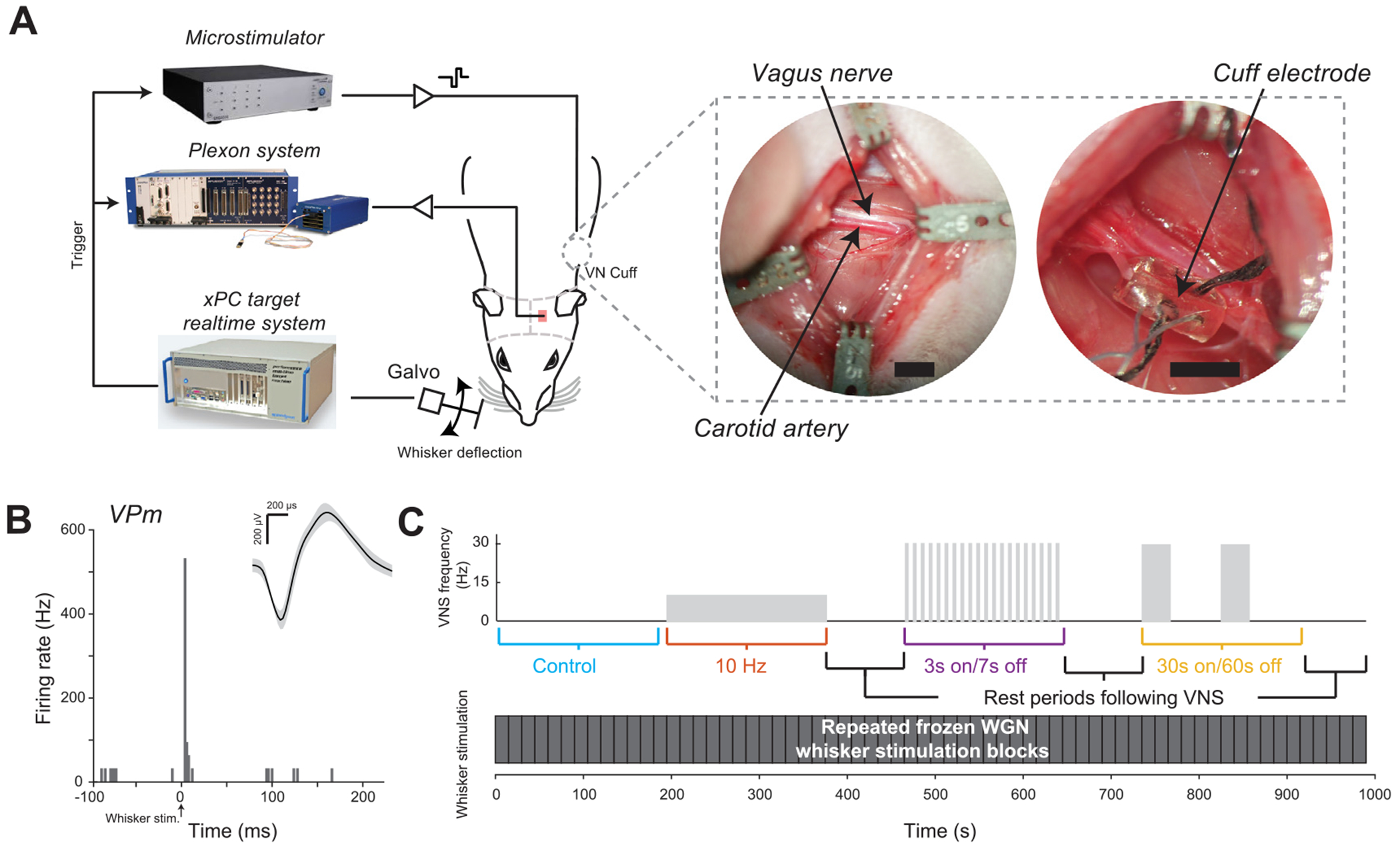
Experimental setup. (A) Diagram of experimental setup and example VNS electrode cuff implantation. (B) Example VPm neuron response to punctate stimulation of its principal whisker. (C) Whisker and VNS stimulation patterns.

To understand the extent to which VNS modulates thalamic sensory processing, we recorded single-unit activity from the VPm of the rat vibrissa pathway in response to repeated WGN whisker deflection while systematically varying VNS stimulation patterns (figure 1(a)). The VPm is a relay nucleus in the thalamus that gates somatosensory information to the cortex (Diamond et al 2008, Millard et al 2013). VPm neurons reliably respond to stimulation of the neuron’s corresponding principle whisker (Wang et al 2010, 2012) (figure 1(b)). Four different VNS patterns were tested: no stimulation (as a control), standard duty-cycle (30 Hz, 30 s on/60 s off duty-cycle), continuous tonic (10 Hz), and fast duty-cycle (30 Hz, 3 s on/7 s off duty-cycle) (figure 1(c)). Each VNS pattern lasted 180 s, during which 15 repetitions of the frozen 15 s WGN whisker stimulation were delivered, with at least 75 s of rest period between them.

### Standard duty-cycle VNS improved thalamic feature selectivity and information transmission

**Figure 2.**
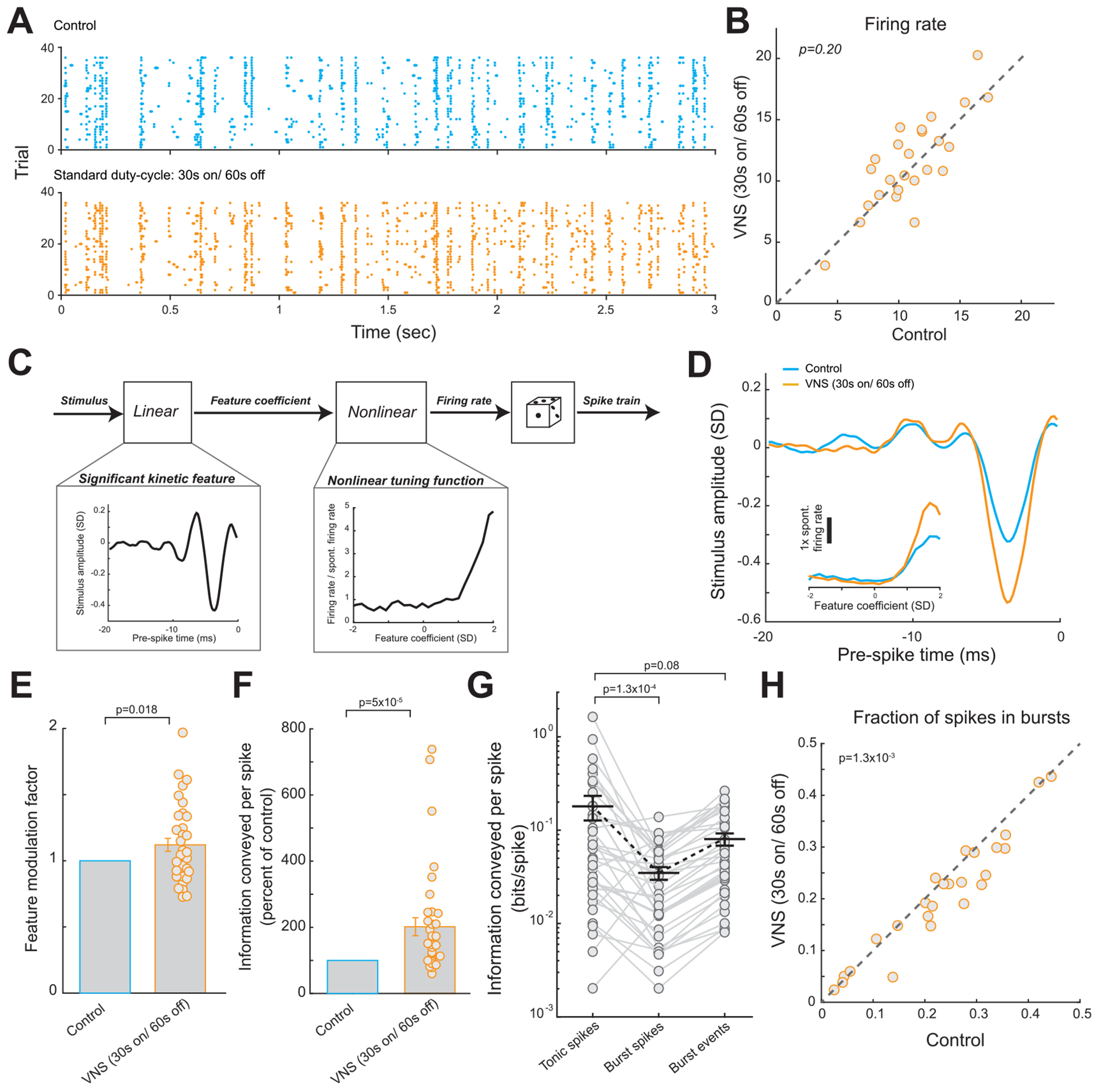
Standard duty cycle VNS improved thalamic feature selectivity and information transmission while suppressing burst firing. (A) Spike raster plot of an example VPm response to repeated presentation of the same white Gaussian noise (WGN) whisker stimulation. (B) Firing rate of VPm neurons to the same WGN whisker stimulation with and without VNS. (C) Linear-nonlinear-poisson model used for white noise reverse correlation analysis. (D) The kinetic feature encoded by an example VPm neuron recovered with and without VNS, inset: corresponding nonlinear tuning functions. (E) Summary of feature modulation factor with and without VNS. (F) Summary of improvement in information transmission efficiency by VNS. (G) Summary plot of information conveyed by tonic spikes, burst spikes, and burst events. (H) Summary of percent of thalamic spikes in bursts with and without VNS. Error bars indicate SEM.

To estimate the feature selectivity of VPm neurons and the effects of VNS on thalamic sensory processing, for each VPm neuron we compared its response to the same frozen white Gaussian noise (WGN) whisker stimulation with and without VNS. The striations clearly visible in the raster plots of recorded VPm spiking activity in response to repeated presentations of the same WGN stimulation indicated that the neurons were sensitive to certain kinetic features in the stimulus, as the cells reliably fired at certain time points during each presentation (figure 2(a)). Standard duty-cycle VNS (i.e. 30 Hz, 30 s on/60 s off) did not change the firing rate of the thalamic relay neurons (figure 2(b); 11.0 ± 0.6 Hz during control periods versus 11.5 ± 0.7 Hz during standard duty-cycle VNS, 25 neurons, 6 rats, *p* = 0.20, paired *t*-test; Mean ± SEM reported for all results unless otherwise stated). However, by using spike triggered covariance analysis to assess the selectivity of the response of the VPm neurons to specific kinetic features (Petersen et al 2008, Rodenkirch et al 2019) (figure 2(c)), we found that VNS improved the feature selectivity of VPm neurons, indicated by an increase in the amplitude of the recovered features which the neurons selectively responded to and the tilting up of nonlinear tuning function at high feature coefficient values (Rodenkirch et al 2019) (figure 2(d)). As the magnitude of the feature coefficient at any given time point represents the similarity between the stimulus and a feature, this alteration in the shape of the nonlinear tuning function indicates an increased selectivity of the neuron to only spike following stimuli that closely match the neuron’s encoded feature. To quantitatively measure the change in the amplitude of the recovered features, we used a feature modulation factor as previously defined (Rodenkirch et al 2019) (**see** Methods). A feature modulation factor of 1 suggests that there was no significant change in encoded kinetic features, whereas a value greater than 1 suggests an increase in amplitude without a change in shape. Standard duty-cycle VNS was found to result in feature modulation factors significantly larger than 1 (figure 2(e), 1 without VNS versus 1.21 ± 0.05 during standard duty-cycle VNS, 36 features, 25 neurons, 6 rats, *p* = 1.8 × 10^−2^, paired *t*-test).

To quantify the effects of VNS on both the encoded kinetic features and nonlinear tuning functions for each neuron, we employed an information theoretic approach to estimate the information transmitted by each VPm spike about the presence/absence of the encoded feature in the stimulus (Rodenkirch et al 2019). Consistent with observations of improved feature selectivity, we found standard duty-cycle VNS dramatically increased both information transmission efficiency (figure 2(f), 202% ± 27% of control bits/spike with standard duty-cycle VNS, 36 features, 25 neurons, 6 rats, *p* = 5.0 × 10^−5^, Wilcoxon signed-rank test; supplementary figure 1(a) (stacks.iop.org/JNE/17/026027/mmedia), 0.13 ± 0.03 bits/spike without VNS versus 0.20 ± 0.05 bits/spike with standard duty-cycle VNS, 36 features, 25 neurons, 6 rats, *p* = 4.6 × 10^−4^) and information transmission rate (supplementary figure 1(b), 206% ± 28% of control bits/second with standard duty-cycle VNS, 36 features, 25 neurons, 6 rats, *p* = 1.4 × 10^−6^, Wilcoxon signed-rank test).

Consistent with previous work, we also observed thalamic relay neurons exhibited burst firing under control conditions (Rodenkirch et al 2019, Sherman 1996). Since thalamic bursts have been linked to deterioration of transmission of information about detailed stimulus features (Sherman 2001, Wolfart et al 2005), we hypothesized that VNS-induced enhancement of sensory processing might also coincide with suppressed burst firing of VPm neurons. Our data showed that thalamic burst spikes did not transmit as much information as tonic spikes (figure 2(g), 0.18 ± 0.05 bits/spike with tonic spikes versus 0.035 ± 0.005 bits/spike with burst spikes, without VNS, 36 features, 25 neurons, 6 rats, *p* = 1.3 × 10^−4^, Wilcoxon signed-rank test). When comparing the information transmitted by tonic spikes to that transmitted by each burst when considered as a point event, we found that burst events on average transmitted less information than tonic spikes (figure 2(g), 0.18 ± 0.05 bits/spike with tonic spikes versus 0.080 ± 0.01 bits/spike with burst events, without VNS, 36 features, 25 neurons, 6 rats, *p* = 0.08, Wilcoxon signed-rank test). However, the difference was not quite significant, most likely due to limited sampling. As we expected, VNS decreased the fraction of VPm spikes in bursts (figure 2(h), 23% ± 2% without VNS versus 21% ± 2% during standard duty-cycle VNS, 25 neurons, 6 rats, *p* = 1.3 × 10^−3^, paired *t*-test).

To ensure the system had ample time to reset to baseline conditions during the rest periods interleaved between VNS conditions, we compared each VPm neuron’s response during the control time period without VNS stimulation to the same neuron’s response occurring during the second half of all of the rest periods (45–75 s after the cessation of the preceding VNS condition). Confirming our correct experimental design, we found the effects of VNS on sensory processing were transient and dissipated within 60 s of cessation of VNS. This was quantitatively confirmed as we found no significant difference in feature modulation (supplementary figure 2(a), 1 during control period versus 0.96 ± 0.04 during second half of rest periods, 36 features, 25 neurons, 6 rats, *p* = 0.27, paired *t*-test), the percent of spikes in bursts (supplementary figure 2(b), 23% ± 2% during control period versus 24% ± 2% during second half of rest periods, 25 neurons, 6 rats, *p* = 0.48, paired t-test), and information transmission (supplementary figure 2(c), 0.13 ± 0.03 bits/spike during control period versus 0.14 ± 0.04 bits/spike during second half of rest periods, 36 features, 25 neurons, 6 rats, *p* = 0.21, Wilcoxon signed-rank test). These results suggest that, unlike previously reported VNS-induced effects which are neuroplasticity-based and last over long timescales, VNS enhancement of sensory processing rapidly dissipates following cessation of VNS. Further, this confirms that the periods of rest time we inserted between VNS conditions were long enough to allow for the system to return to baseline conditions.

### VNS-induced improvement of thalamic information transmission is similar to that induced by direct LC activation

**Figure 3.**
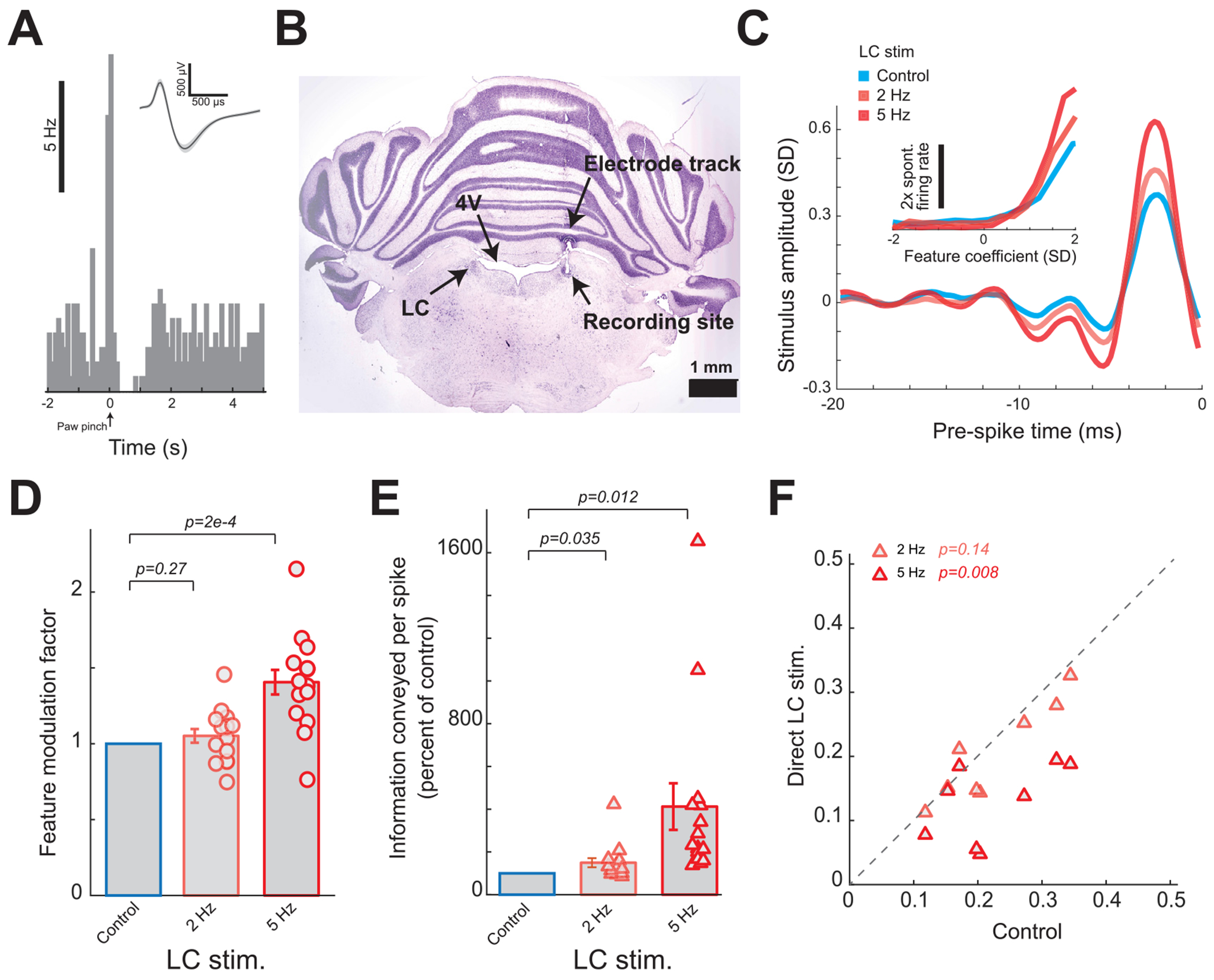
Direct LC activation increased thalamic information transmission in ketamine-anesthetized rats. (A) Example LC phasic response to paw pinches followed by an inhibititory period; inset: wide spike waveform of the corresponding LC cell (shaded area indicates S.D.). (B) Histological confirmation of correct electrode placement in the LC. (C) Feature selectivity of an example VPm neuron recovered with and without LC stimulation; inset: corresponding nonlinear tuning function. (D) Summary of feature modulation factor during varying LC stimulation patterns. (E) Summary of improvement of information transmission (bits/spike) during varying LC stimulation patterns. (F) Summary of thalamic burst firing under varying patterns of LC stimulation. Error bars indicate SEM.

Our recent work demonstrated that direct LC activation improved thalamic feature selectivity and information transmission through regulating thalamoreticulo-thalamic circuit dynamics in pentobarbital-anesthetized rats. Here we observed that VNS produced similar effects as those we observed in result to direct LC stimulation, specifically VNS both improved thalamic feature selectivity and information transmission while decreasing thalamic burst firing. As a recent study demonstrated the causal link between VNS and LC activity (Hulsey et al 2017), we wanted to confirm that direct LC stimulation in ketamine-anesthetized rats would produce similar effects as we observed with VNS. To this end, we measured thalamic feature selectivity and information transmission with and without direct LC activation in rats under ketamine anesthesia. LC neurons were identified based on their wide spike waveform, phasic response to paw pinch followed by inhibition (Liu et al 2017), and electrode placement in the LC which was histologically verified on a subset of recordings (figures 3(a) and (b)). After electrophysiologically confirming the position of the electrode within the LC, we disconnected the recording system and connected an electrical microstimulator to the electrode. Similar to our previous work, we found both direct 2 Hz and 5 Hz LC stimulation significantly improved the feature selectivity, as shown qualitatively by the change in the recovered feature and nonlinear tuning function (figure 3(c)) and measured quantitively by the feature modulation factor (figure 3(d), 1 without LC stimulation versus 1.05 ± 0.05 during 2 Hz LC stimulation or 1.41 ± 0.08 during 5 Hz LC stimulation,15 features across 8 neurons across 3 rats, *p* = 0.27 and 1.9 × 10^−4^ respectively, paired *t*-test). Consequently, this improvement in feature selectivity translated to an improvement in information transmission efficiency (figure 3(e), 150% ± 21% of control bits/spike during 2 Hz LC stimulation or 412% ± 109% of control bits/spike during 5 Hz LC stimulation,15 features across eight neurons across 3 rats, *p* = 3.5 × 10^−2^ and 1.2 × 10^−2^ respectively, paired *t*-test; supplementary figure 3(a), 0.15 ± 0.08 bits/spike without LC stimulation versus 0.21 ± 0.11 bits/spike with 2 Hz LC stimulation and 0.39 ± 0.21 bits/spike with 5 Hz LC stimulation, 15 features, eight neurons, 3 rats, *p* = 3.4 × 10^−3^ and 6.1 × 10^−5^ respectively, Wilcoxon signed-rank test) and rate (supplementary figure 3(b), 153% ± 22% of control bits/sec during 2 Hz LC stimulation or 337% ± 81% of control bits/sec during 5 Hz LC stimulation, 15 features across eight neurons across 3 rats, *p* = 2.9 × 10^−2^ and 1.1 × 10^−2^ respectively, paired *t*-test). Importantly, direct LC stimulation in ketamine anesthetized rats also significantly suppressed burst firing, as indicated by a significant reduction in the fraction of spikes in bursts (figure 3(f), 22% ± 3% without LC stimulation versus 20% ± 3% during 2 Hz LC stimulation or 13% ± 2% during 5 Hz LC stimulation, eight neurons across 3 rats, *p* = 0.14 and 7.4 × 10^−3^ respectively, paired *t*-test). Taken together, these results suggest that VNS modulates thalamic sensory processing at least partially through the LC-NE system (see Discussion).

### The short timescale of VNS effects on thalamic sensory processing caused standard duty-cycle patterns of VNS to induce a fluctuating thalamic sensory processing state

**Figure 4.**
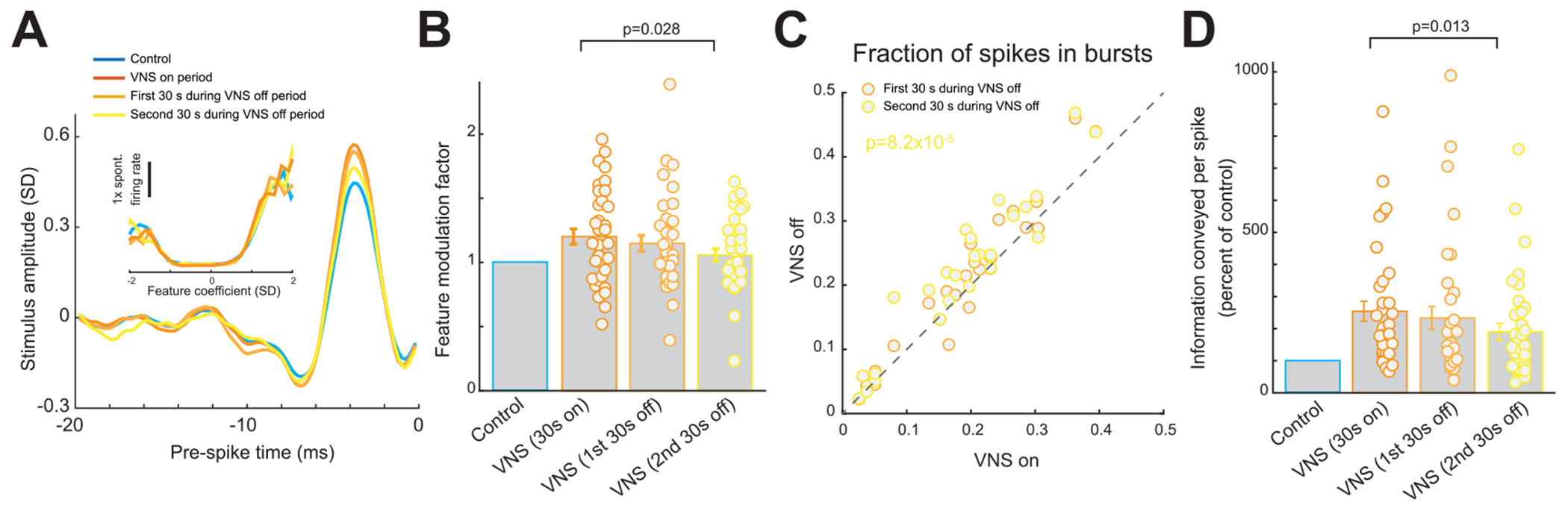
Standard duty-cycle VNS induced a fluctuating thalamic sensory processing state. (A) Feature selectivity of an example VPm neuron recovered during the on versus second half of off period of standard duty-cycle VNS; inset: corresponding nonlinear tuning function. (B) Summary of feature modulation factor during the different periods of standard duty-cycle VNS. (C) Summary of burst suppression during the different periods of standard duty-cycle VNS. (D) Summary of improvement in information transmission during the different periods of standard duty-cycle VNS. Error bars indicate SEM.

A typical therapeutically employed VNS stimulation pattern traditionally uses a relatively slow duty-cycle (e.g. 30 s on/60 s off). Importantly, the off period of the standard VNS pattern used in this paper (60 s) is longer than the period we found it takes for the effects of VNS on sensory processing to dissipate (~45 s). Although relatively slow duty-cycled patterns have proved to efficiently mitigate symptoms in neurological disorders, it was unclear how switching VNS on and off would modulate thalamic state given that the effects of VNS on VPm sensory processing occur and dissipate on such short timescales. To test this, we compared the responses of VPm neurons during the on period of VNS to the same neurons’ responses during the first 30 s and second 30 s of the off period. Interestingly, we found that the effect of VNS on thalamic feature selectivity and information transmission rapidly diminished during the off period. The amplitude of the recovered encoded features was significantly smaller during the second 30 s of the VNS off period than during the VNS on period (figure 4(a)). Quantifying this difference in recovered feature amplitude using the feature modulation factor, we found that the factor was larger during the on sections than the off sections of the standard duty-cycle VNS (figure 4(b), 1.20 ± 0.06 during on period versus 1.06 ± 0.05 during second half of off period, 36 features, 25 neurons, 6 rats, *p* 2.8 × 10^−3^, paired *t*-test). The fluctuations in thalamic processing state induced by standard duty-cycle VNS were further evidenced by the observation that there was a significant change in percent of spikes in bursts in the second 30 s of the VNS off period as compared to the VNS on period (figure 4(c), 19 ± 2% during on period versus 22% ± 2% during second half of off period, 25 neurons, 6 rats, *p* = 8.2 × 10^−5^, paired *t*-test). Accordingly, the information transmitted per spike was significantly less during the second half of the off period than the on period of the standard duty-cycle VNS (figure 4(d), 254% ± 31% of control bits/spike during on period versus 190% ± 26% of control bits/spike during second half of off period, 36 features, 25 neurons, 6 rats, *p* = 1.3 × 10^−2^, paired *t*-test). Taken together, these results indicate that standard duty-cycle VNS created a fluctuating state of sensory processing in the thalamus. Here we predict that this fluctuating state would be sub-optimal for perceptual sensitivity, as the same stimulus occurring during the on period of the VNS cycle would evoke a different thalamic response than if it occurred during the off period of the VNS cycle and therefore may be incorrectly perceived as a different stimulus.

### Fast duty-cycle VNS enhanced thalamic information transmission without inducing fluctuations

Our data have shown that as VNS rapidly induced improvement in thalamic sensory processing, and that this improvement quickly faded away once VNS was turned off, standard duty-cycle VNS patterns resulted in a fluctuating thalamic sensory processing state. A possible way to achieve the benefits of VNS on thalamic sensory processing without inducing a fluctuating state would be to use fast duty-cycle VNS (e.g. 3 s on/7 s off) or continuous tonic VNS, both of which do not have long off periods. To assess whether these stimulation patterns could be used for optimal, fluctuation-free enhancement of sensory processing, we performed standard duty-cycle (30 s on 60 s off), fast duty-cycle (3 s on 7 s off), and continuous (10 Hz) VNS in the same recording session and compared the effects of the various VNS patterns on thalamic feature selectivity.

**Figure 5.**
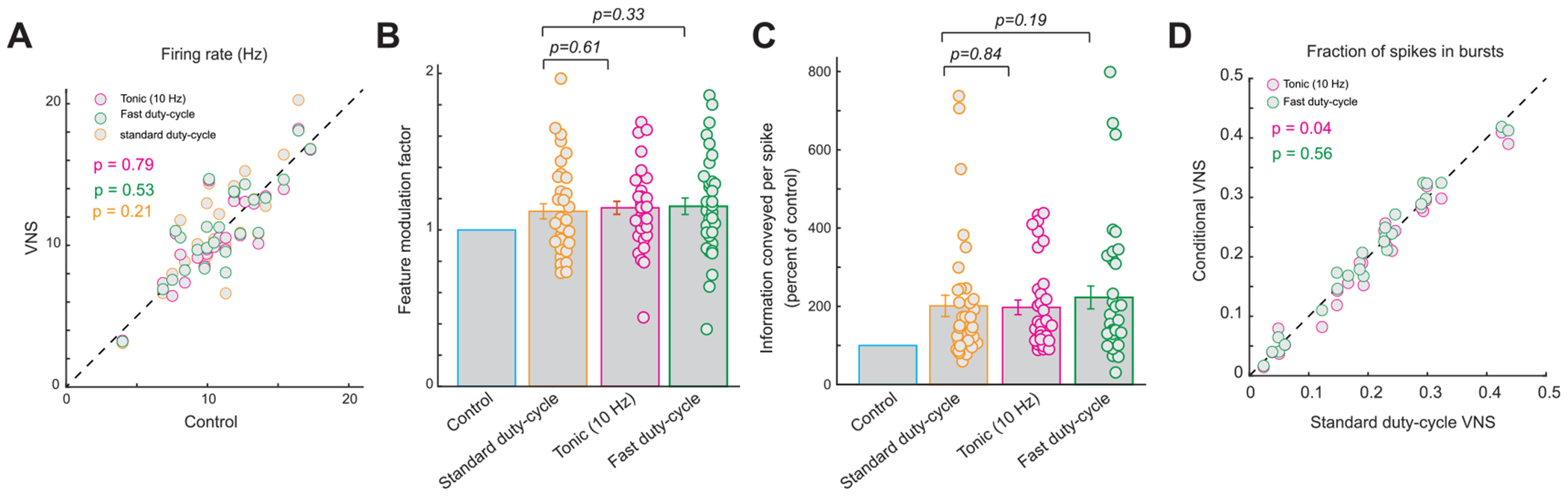
Fast duty-cycle and tonic VNS induced similar improvement in thalamic information transmission as observed with standard duty-cycle VNS. (A) Summary of VPm firing rate in response to the same whisker stimulation during the varying patterns. (B) Summary of feature modulation factor during the different VNS patterns. (C) Summary of improvement in information transmission efficiency during the different VNS patterns. (D) Summary of fraction of spikes in bursts during the different VNS patterns. Error bars indicate SEM.

None of the three VNS patterns resulted in a significantly different VPm firing rate as compared to control conditions (figure 5(a), 11.0 ± 0.6 Hz without VNS versus 10.9 ± 0.7 Hz during 10 Hz tonic VNS, 11.2 ± 0.7 Hz during fast duty-cycle VNS, and 11.6 ± 0.7 Hz during standard duty-cycle VNS, 25 neurons, 6 rats, *p* = 0.79, 0.53 and 0.21 respectively, paired *t*-test). Further, we found that all three conditions produced similar improvements in thalamic feature selectivity as quantified by the feature modulation factor (figure 5(b), 1.12 ± 0.05 during standard duty-cycle VNS versus 1.14 ± 0.04 during 10 Hz tonic VNS or 1.15 ± 0.05 during fast duty-cycle VNS, 36 features, 25 neurons, 6 rats, *p* = 0.61 and 0.33, respectively, paired *t*-test) and information transmission efficiency (figure 5(c), 202% ± 27% of control bits/spike during standard duty-cycle VNS versus 197% ± 19% of control bits/spike during 10 Hz tonic VNS or 223% ± 29% of control bits/spike during fast dutycycle VNS, 36 features, 25 neurons, 6 rats, *p* = 0.84 and 0.19, respectively, paired *t*-test; supplementary figure 4, 0.20 ± 0.05 bits/spike during standard dutycycle VNS versus 0.18 ± 0.04 bits/spike during 10 Hz tonic VNS and 0.20 ± 0.05 bits/spike during fast dutycycle VNS, 36 features, 25 neurons, 6 rats, *p* = 0.77 and 0.53, Wilcoxon signed-rank test and paired *t*-test, respectively). Further, all VNS patterns produced a VPm response with a similar percent of spikes in bursts (figure 5(d), 21% ± 2% during standard duty-cycle VNS versus 20% ± 2% during 10 Hz tonic VNS or 21% ± 2% during fast duty-cycle VNS, 25 neurons, 6 rats, *p* = 0.04 and 0.56, respectively, paired *t*-test), with all VNS patterns resulting in a decrease in the percent of spikes in bursts when compared to control conditions.

**Figure 6.**
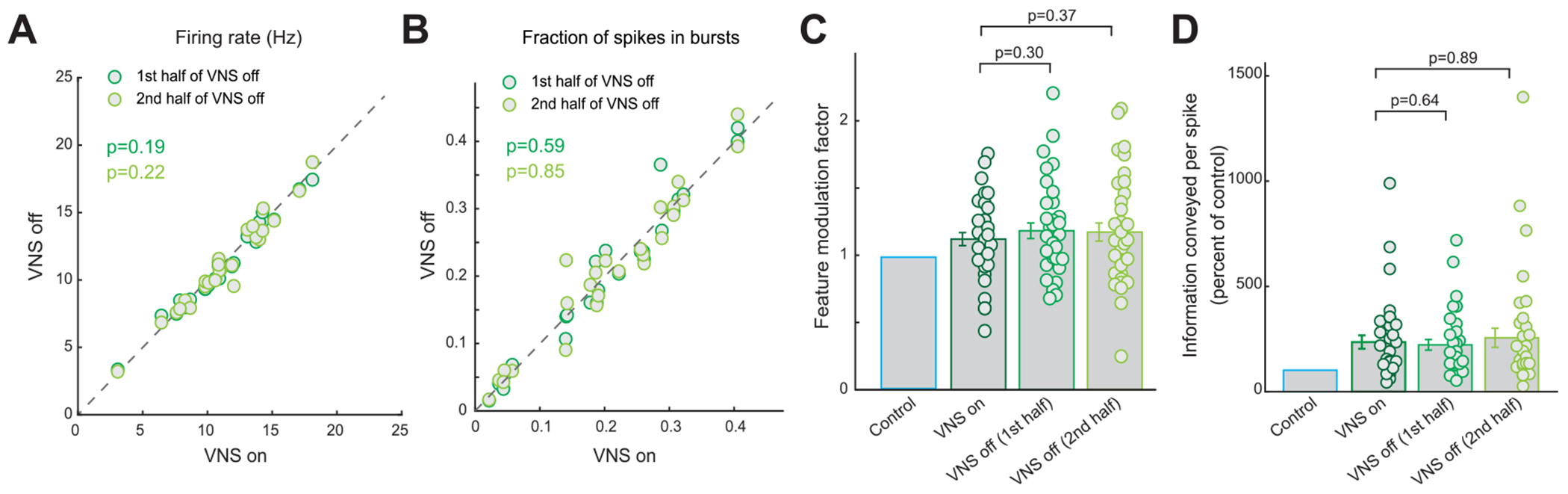
Fast duty-cycle VNS caused no fluctuation in improvement of thalamic information transmission. (A) Summary of firing rate during the different periods of fast duty-cycle VNS. (B) Summary of feature modulation factor during the different periods of fast duty-cycle VNS. (C) Summary of burst suppression during the different periods of fast duty-cycle VNS. (D) Summary of improvement of information transmission during the different periods of fast duty-cycle VNS. Error bars indicate SEM.

We next investigated whether fast duty-cycle VNS introduced any fluctuations in VPm sensory processing state similar to those observed to be induced by standard duty-cycle VNS. In a similar fashion as to our analysis of the different stages of the standard duty-cycle, we separated the response of the VPm neurons during the on periods of the fast duty-cycle stimulus and compared it with the same neuron’s response during the first or second half of the off period. Here we found no significant difference in firing rate (figure 6(a), 11.3 ± 0.7 Hz during on period versus 11.2 ± 0.7 Hz during first half of off period or 11.1 ± 0.7 Hz during second half of off period, 25 neurons, 6 rats, *p* = 0.19 and 0.22 respectively, paired *t*-test) and percent of spikes in bursts (figure 6(b), 21% ± 2% during on period versus 21% ± 2% during first half of off period or 21% ± 2% during second half of off period, 25 neurons, 6 rats, *p* = 0.59 and 0.85 respectively, paired t-test) between the on period of fast duty-cycle VNS and the first half or second half of the off cycle.

More importantly, both the improvement in feature selectivity and change in nonlinear tuning function did not fluctuate between the on period and first half and second half of the off periods of fast duty-cycle VNS. This lack of fluctuation in feature selectivity during fast duty-cycle VNS translated to no difference in the feature modulation factor between the on period and either half of the off period (figure 6(c), 1.12 ± 0.05 during on period versus 1.18 ± 0.06 during first half of off period or 1.17 ± 0.07 during second half of off period, 36 features, 25 neurons, 6 rats, *p* = 0.30 and 0.37 respectively, paired *t*-test). Further, we find no difference in the strength of improvement of information transmission efficiency between the on period and either half of the off periods of fast duty-cycle VNS (figure 6(d), 236% ± 32% of control bits/spike during on period versus 223% ± 25% of control bits/spike during first half of off period or 256% ± 45% of control bits/spike during second half of off period, 36 features, 25 neurons, 6 rats, *p* = 0.64 and 0.89, respectively, paired t-test and Wilcoxon signed-rank test, respectively). Together, these results indicate that both fast duty-cycle VNS and tonic VNS result in the same level of improvement in thalamic sensory processing as standard duty-cycle VNS, without inducing a fluctuating thalamic sensory processing state that was induced by standard dutycycle VNS. This is important as during a fluctuating thalamic sensory processing state, the same stimulus would evoke a different thalamic response if received at different time points in the fluctuation which may degrade the ability to discriminate between similar stimuli.

### The effects of fast duty-cycle and tonic VNS on thalamic sensory processing were amplitude dependent

Our results have suggested that both fast duty-cycle and tonic VNS could be used to optimally enhance thalamic sensory processing whereas standard duty-cycle VNS is suboptimal for this purpose as it induces fluctuations in thalamic processing state. During the experiments which compared the effects of these stimulation patterns, all VNS pulses were delivered at a fixed current amplitude of 1 mA. However, the amplitude of VNS being currently used in clinical situations can vary from patient to patient and exists within a wide range of values (Heck et al 2002, Musselman et al 2018, Yamamoto 2015). More importantly, it has been found that some effects of VNS have an inverted U shape relationship with VNS amplitude (Clark et al 1995, Clark et al 1999, Clark et al 1998, Zuo et al 2007, Revesz et al 2008). Therefore, we wanted to determine the effects of different amplitudes of VNS on sensory processing. To this end, we carried out new experiments to examine the sensitivity of VNS effects on thalamic information transmission to VNS amplitude. We compared four different VNS amplitudes: 0 (as a control), 0.4 mA, 1 mA, and 1.6 mA.

**Figure 7.**
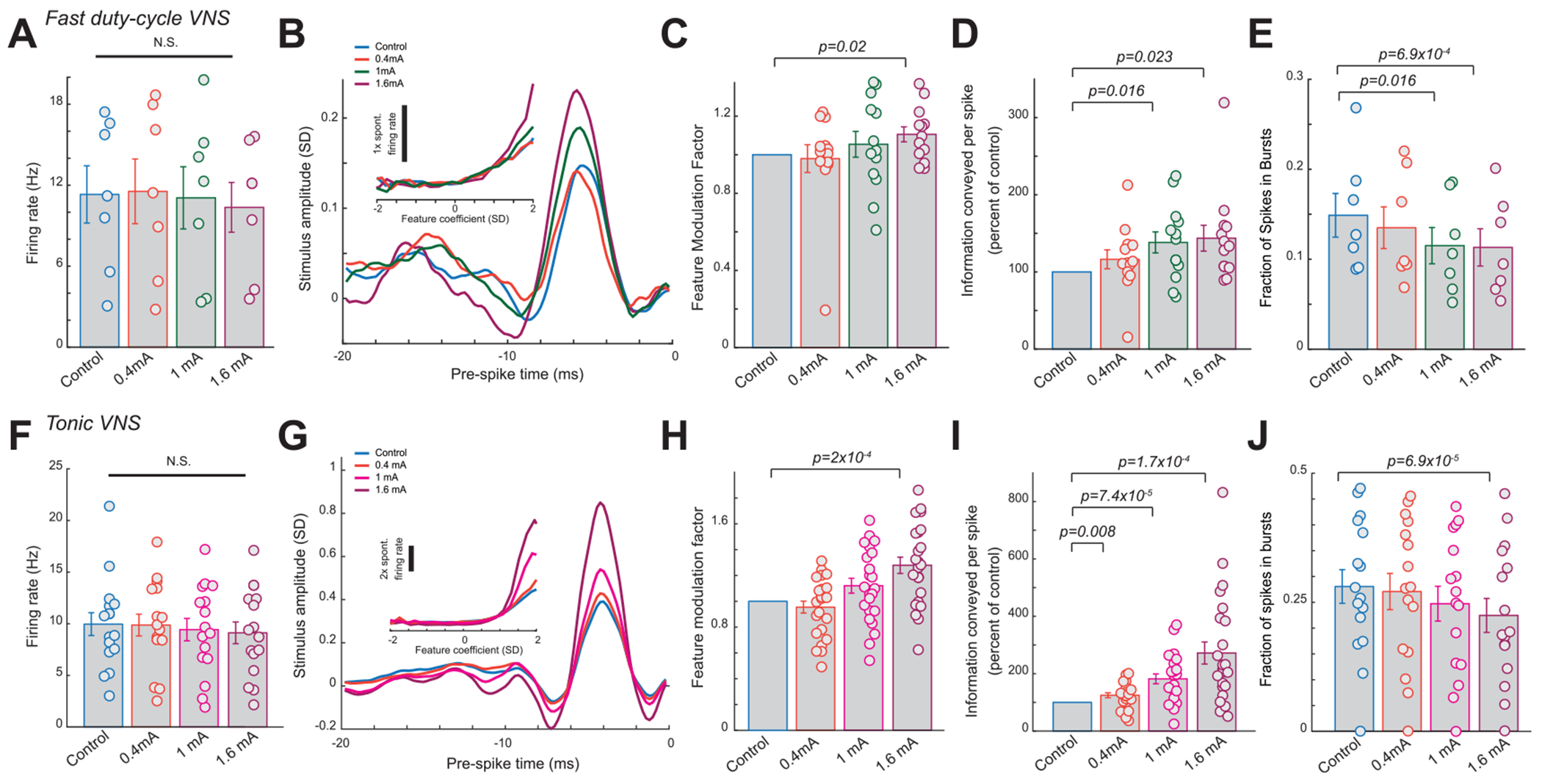
The strength of the effects of fast duty-cycle and tonic VNS on improvement of thalamic sensory increase monotonically with increasing VNS amplitude. (A) Summary of VPm firing rate during varying amplitudes of fast duty-cycle VNS. (B) Feature selectivity of an example VPm neuron recovered during varying amplitudes of fast duty-cycle VNS; inset: corresponding nonlinear tuning function. (C) Summary of feature modulation factor during varying amplitudes of fast duty-cycle VNS. (D) Summary of improvement in information transmission during varying amplitudes of fast duty-cycle VNS. (E) Summary of fraction of spikes in bursts during varying amplitudes of fast duty-cycle VNS. (F) Summary of VPm firing rate during varying amplitudes of tonic VNS. (G) Feature selectivity of an example VPm neuron recovered during varying amplitudes of tonic VNS; inset: corresponding nonlinear tuning function. (H) Summary of feature modulation factor during varying amplitudes of tonic VNS. (I) Summary of improvement in information transmission during varying amplitudes of tonic VNS. (J) Summary of fraction of spikes in bursts during varying amplitudes of tonic VNS. Error bars indicate SEM.

When analyzing fast duty-cycle VNS at different amplitudes, we found none of the three amplitudes induced changes in VPm firing rate in response to WGN whisker stimulation as compared to the control period (figure 7(a), 11.3 ± 2.1 Hz during control without VNS versus 11.6 ± 2.4 Hz during 0.4 mA fast dutycycle VNS, 11.1 ± 2.3 Hz during 1 mA fast duty-cycle VNS, and 10.36 ± 1.8 Hz during 1.6 mA fast dutycycle VNS, 7 neurons, 2 rats, *p* = 0.65, 0.80, and 0.21, respectively, paired *t*-test). However, we found that the strength of fast duty-cycle VNS-induced improvements in feature selectivity and information transmission monotonically increased with fast duty-cycle VNS amplitude (figure 7(b)) as quantitatively measured by the feature modulation factor (figure 7(c), 1 during control without VNS versus 0.98 ± 0.07 during 0.4 mA fast duty-cycle VNS, 1.05 ± 0.07 during 1 mA fast duty-cycle VNS, or 1.11 ± 0.04 during 1.6 mA fast duty-cycle VNS, 13 features, seven neurons, 2 rats, *p* = 0.78, 0.44, and 0.02, respectively, paired t-test) and information transmission efficiency (figure 7(d), 116% ± 12% of control bits/spike during 0.4 mA fast duty-cycle VNS, 138% ± 14% of control bits/spike during 1 mA fast duty-cycle VNS, or 144% ± 17% of control bits/spike during 1.6 mA fast duty-cycle VNS, 13 features, seven neurons, two rats, *p* = 0.20, 1.6 × 10^−2^, and 2.3 × 10^−2^ respectively, paired t-test). As expected, burst firing also decreased monotonically with the increase in fast duty-cycle VNS amplitude as evidenced by a decrease in the percent of spikes in bursts (figure 7(e), 14.9% ± 2.4% during control without VNS versus 13.5% ± 2.3% during 0.4 mA fast duty-cycle VNS, 11.5% ± 2.0% during 1 mA fast duty-cycle VNS, or 11.3% ± 2.0% during 1.6 mA fast duty-cycle VNS, seven neurons, two rats, *p* = 0.17, 1.63 × 10^−2^, and 6.93 × 10^−4^, respectively, paired *t*-test).

Similarly, when analyzing 10 Hz tonic VNS at different amplitudes, we found none of the three amplitudes induced changes in VPm firing rate in response to WGN whisker stimulation as compared to the control period (figure 7(f), 10.0 ± 1.1 Hz during control without VNS versus 9.9 ± 1.1 Hz during 0.4 mA 10 Hz VNS, 9.4 ± 1.1 Hz during 1 mA 10 Hz VNS, and 9.1 ± 1.1 Hz during 1.6 mA 10 Hz VNS, 16 neurons, five rats, *p* = 0.84, 0.46, and 0.08 respectively, paired *t*-test). We also found that the strength of tonic VNS-induced improvements in feature selectivity and information transmission efficiency monotonically increased with tonic VNS amplitude (figure 7(g)) as quantitatively measured by the feature modulation factor (figure 7(h), 1 during control without VNS versus 0.95 ± 0.05 during 0.4 mA 10 Hz VNS, 1.12 ± 0.06 during 1 mA 10 Hz VNS, or 1.28 ± 0.06 during 1.6 mA 10 Hz VNS, 24 features, 16 neurons, five rats, *p* = 0.33, 0.048, and 2.03 × 10^−4^ respectively, paired t-test) and information transmission efficiency (figure 7(i), 125% ± 8% of control bits/spike during 0.4 mA 10 Hz VNS, 182% ± 17% of control bits/spike during 1 mA 10 Hz VNS, or 272% ± 38% of control bits/spike during 1.6 mA 10 Hz VNS, 24 features, 16 neurons, five rats, *p* = 7.53 × 10^−3^, 7.43 × 10^−5^, and 1.73 × 10^−4^ respectively, paired t-test). As expected, burst firing also decreased monotonically with increasing tonic VNS amplitude as evidenced by a decrease in the percent of spikes in bursts (figure 7(j), 28.1% ± 3.3% during control without VNS versus 27.1% ± 3.5% during 0.4 mA 10 Hz VNS, 24.7% ± 3.4% during 1 mA 10 Hz VNS, or 22.5% ± 3.3% during 1.6 mA 10 Hz VNS, 16 neurons, five rats, *p* = 0.36, 5.63 × 10^−2^, and 6.93 × 10^−5^ respectively, paired *t*-test). Taken together, these characterization results suggest that VNS rapidly improves thalamic sensory processing in an amplitude dependent fashion.

### The effects of VNS on thalamic sensory processing were frequency dependent

VNS with different frequencies can have distinguishable effects in clinical applications (Heck et al 2002, Musselman et al 2018, Yamamoto 2015). Therefore, we wanted to evaluate how different frequencies of VNS affect thalamic sensory processing. To this end we compared the responses of VPm neurons during 10 Hz, 1 mA continuous tonic VNS to the same neurons’ responses during 30 Hz, 1 mA continuous tonic VNS (taken from the on periods of the standard duty-cycle VNS).

**Figure 8.**
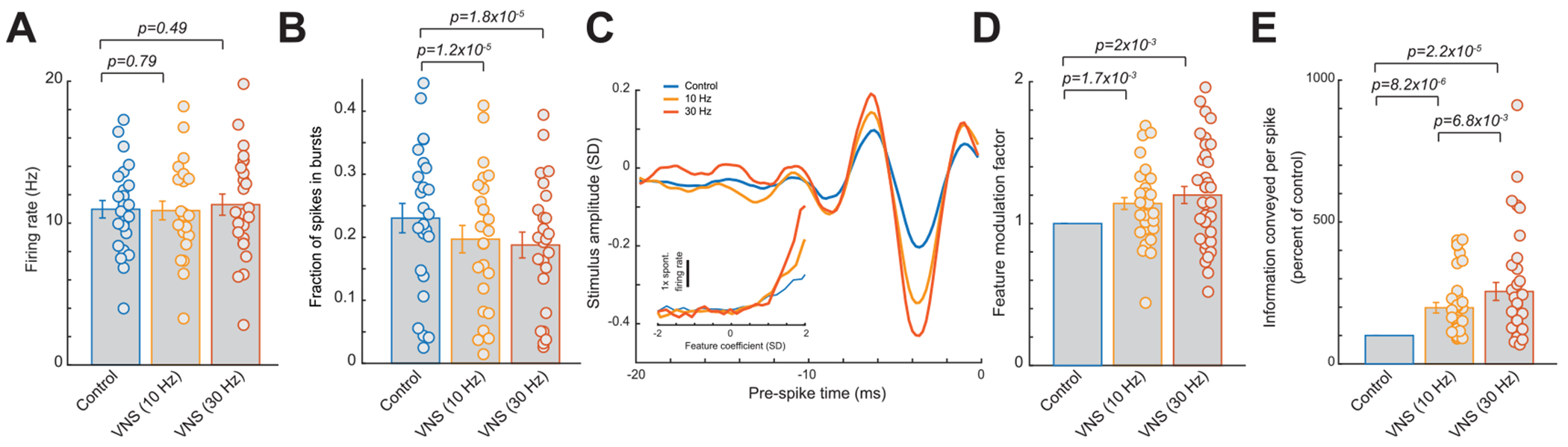
The strength of the effects of tonic VNS on improvement of thalamic sensory increase monotonically with increasing VNS frequency. (A) Summary of VPm firing rate during varying frequencies of tonic VNS. (B) Summary of fraction of spikes in bursts during varying frequencies of tonic VNS. (C) Feature selectivity of an example VPm neuron recovered during varying frequencies of tonic VNS; inset: corresponding nonlinear tuning function. (D) Summary of feature modulation factor during varying frequencies of tonic VNS. (E) Summary of improvement in information transmission during varying frequencies of tonic VNS. Error bars indicate SEM.

Again, we found that both frequencies of tonic VNS resulted in firing rates that were not significantly different than during the control period (figure 8(a), 11.0 ± 0.6 Hz during control without VNS versus 10.9 ± 0.7 Hz with 10 Hz VNS or 11.3 ± 0.7 Hz during 30 Hz VNS, 25 neurons, six rats, *p* = 0.79 and 0.49 respectively, paired *t*-test). However, the percent of spikes in bursts decreased monotonically with increasing tonic VNS frequency (figure 8(b), 23.0% ± 2.3% during control without VNS versus 19.4% ± 2.2% with 10 Hz VNS or 18.8% ± 2.0% during 30 Hz VNS, 25 neurons, six rats, *p* = 1.23 × 10^−5^ and 1.8 × 10^−5^ respectively, paired t-test). Moreover, we found that 30 Hz VNS produced a stronger increase in recovered feature amplitude and tilting up of the nonlinear tuning function. When we quantified the effects of 10 Hz and 30 Hz tonic VNS on the recovered features, we observed that both produced a significantly larger feature modulation factor than 1, which increased monotonically with increasing tonic VNS frequency (figures 8(c) and (d), 1 during control without VNS versus 1.14 ± 0.04 during 10 Hz VNS or 1.20 ± 0.06 during 30 Hz VNS, 36 features, 25 neurons, six rats, *p* = 1.7 × 10^−3^ and 1.9 × 10^−3^ respectively, paired *t*-test). Consequently, due to VNS effects on sensory processing increasing monotonically with tonic VNS frequency, we found the information transmission efficiency also monotonically increased with tonic VNS frequency (figure 8(e), 198% ± 19% of control bits/spike during 10 Hz VNS versus 255% ± 32% of control bits/spike during 30 Hz VNS, 36 features, 25 neurons, six rats, *p* = 8.2 × 10^−6^ and 2.2 × 10^−5^, respectively, paired t-test). Further we found information transmission efficiency was significantly more strongly improved with 30 Hz VNS than with 10 Hz (figure 8(e), *p* = 6.8 × 10^−3^, paired *t*-test).

## Discussion

Previous work has focused on using VNS to facilitate the neuroplasticity of brain circuits, likely through activation of neuromodulatory systems which are known to induce neuroplasticity (Hays et al 2013). These changes require pairing stimuli or tasks with VNS activation and take place over weeks to months (Buell et al 2019). In contrast, we found that VNS was also able to drastically affect the sensory processing within the thalamus at a short timescale, requiring no prior pairing. Further, we found the effects of VNS on sensory processing to be transient as they dissipated quickly following cessation of VNS. This new application of VNS therefore does not depend on long-term changes induced by neuroplasticity, instead we hypothesize that VNS activation results in rapid, transient regulation of sensory processing in the thalamus most likely through activation of neuromodulation centers that can rapidly change thalamic neurochemical state, such as the LC. We found that VNS-induced improvements of thalamic sensory processing occurred through enhancement of feature selectivity that resulted in an increased efficiency and rate of sensory information transmitted by VPm neurons. Previous studies have shown a causal link between enhanced thalamic sensory processing and improved perceptual performance (Ollerenshaw et al 2014, Rodenkirch et al 2019). Therefore, as our data shows that VNS improves thalamic sensory processing, we predict that certain patterns of VNS could potentially be used to improve behavioral performance in perceptual tasks. Future work is warranted to probe the relationship between different VNS patterns and the enhancement of perceptual performance.

We found that VNS improved thalamic feature selectivity and information transmission in similar fashion as direct LC stimulation. As our previous work demonstrated a causal relationship between LC-stimulation induced suppression of thalamic bursts and improvement in information transmission (Rodenkirch et al 2019), it is important to note that VNS also suppressed burst firing in the thalamus. This is not unexpected as it has been shown that the vagus nerve exerts influence on LC activity through the projection of the NTS and that VNS increases LC activity (Groves et al 2005, Hulsey et al 2017). However, the NTS also projects to other neuromodulatory nuclei in addition to the LC, including the basal forebrain (Lopes et al 2016) which also projects to the sensory thalamus. Activation of either the LC or the basal forebrain has been shown to modulate sensory processing (Goard and Dan 2009, Pinto et al 2013, Rodenkirch et al 2019). Therefore, the improved thalamic sensory processing that we observed here may involve the collective action of multiple neuromodulatory systems activated by VNS. Future work utilizing pharmacological manipulation would be able to tease apart the contribution of the different neuromodulatory systems to the observed VNS-induced improvement in thalamic sensory processing.

In current clinical treatments, VNS is most commonly given in a duty-cycle fashion, such as 30 s on/60 s off (Heck et al 2002, DeGiorgio et al 2005, Yamamoto 2015, Musselman et al 2018), which is based on the assumption that duty-cycled stimulation poses less of a risk of damaging a nerve (Agnew et al 1989). Here we found VNS improvement of thalamic sensory processing is transient and rapidly dissipates following cessation of VNS, which resulted in the effects of VNS dissipating during the off periods of the standard dutycycle VNS. This fluctuating thalamic processing state resulted in VPm neurons exhibiting a difference in feature modulation, sensory information transmission efficiency, and burst firing rate during the on versus the off period of standard duty-cycle VNS. This fluctuating sensory processing state would presumably induce a fluctuating bias in perception that was not related to the stimulus and therefore would act as noise, which may be detrimental to the precise information processing needed during perceptual discrimination tasks. For example, the same stimulus would produce different neural responses if received during the on period versus the off period of the standard duty-cycle, which may cause the same stimuli to be perceived as two different stimuli. Interestingly, we found that VNS with a fast duty-cycle of 3 s on/7 s off did not induce fluctuations in thalamic sensory processing state, presumably due to the fact that the time constants of VNS modulation of sensory processing in the thalamus are faster than those of standard duty-cycle VNS patterns but not those of a fast duty-cycle VNS pattern.

Compromised or abnormal sensory processing, caused by many underlying disorders such as Parkinson’s disease, depression, migraine, central pain syndrome, and ADHD, can strongly impact daily life (Carron et al 2016, Goadsby et al 2017, Serafini et al 2017, Shimizu et al 2014). Relevant to our results here, abnormal thalamic bursting activity has been implicated in the aforementioned disorders (Lenz et al 1988, 1989, Jeanmonod et al 1996, Zirh et al 1998, Ferrarelli and Tononi 2011, Cain and Snutch 2013, Pratt and Morris 2015, Andrade et al 2016). Our results have shown that VNS decreased thalamic bursting suggesting that VNS-induced decrease of thalamic bursting may be one of the mechanisms underlying current VNS-based treatments. Here we found that increasing the frequency of VNS as well as the amplitude of fast duty-cycle VNS and tonic VNS resulted in stronger improvements in sensory processing as evidenced by increased feature selectivity and improved stimulus-related information transmission. Therefore, our results suggest that an optimal state for perceptual processing is best achieved using high frequency and high amplitude VNS delivered either continuously or with a high frequency duty-cycle.

As we have found that high current and frequency patterns of VNS provided the best enhancement of thalamic sensory processing, a pertinent question is how to deliver these aggressive VNS patterns while minimizing risk of vagus nerve damage or patient discomfort. One method would be to use a closed-loop system which engages high amplitude and frequency tonic VNS only during specific time periods, such as when the user expects to receive sensory stimuli or is identified to be in a non-optimal sensory processing state using non-invasive indexes of brain state such as pupil dilation (Liu et al 2017). This type of on-demand VNS-enhancement of sensory processing would be facilitated by the fact that VNS-induced improvements in perception rapidly onset once VNS is initiated. In addition, our previous work suggested that the activation of the LC-NE system is more beneficial during more difficult perceptual tasks (Rodenkirch et al 2019), indicating task-dependent VNS may be an optimal configuration for enhancing behavioral performance.

Newly developed sensory neuroprotheses use patterned microstimulation of different regions along sensory pathways to recover senses lost due to disease, degeneration, or injury (Romo et al 1998, Tehovnik et al 2009, O/’Doherty et al 2011, Bari et al 2013, Kim et al 2015, Rodenkirch et al 2016). The accuracy of the perception induced by these neuroprotheses may be dependent on sensory processing state, as it has been shown that brain-state affects the manner in which information is encoded and processed in these pathways (Panzeri et al 2016, Schriver et al 2018, Rodenkirch et al 2019). Further research exploring the ability of VNS to modulate sensory processing state in such a manner that optimizes it for the writing of patterned microstimulation may improve the ability of brainmachine-interfaces to correctly encode information along sensory pathways.

## Supplementary Material

Supplementary Data

Supplementary material for this article is available online
