# Supplementary material for: Rapid and transient enhancement of thalamic information transmission induced by vagus nerve stimulation
Source: J Neural Eng. Author manuscript; Available in PMC 2026 May 14. (PMC13171290; doi:10.1088/1741-2552/ab6b84)
Supplement: Supplementary Data [file NIHMS2168987-supplement-Supplementary_Data.pdf]

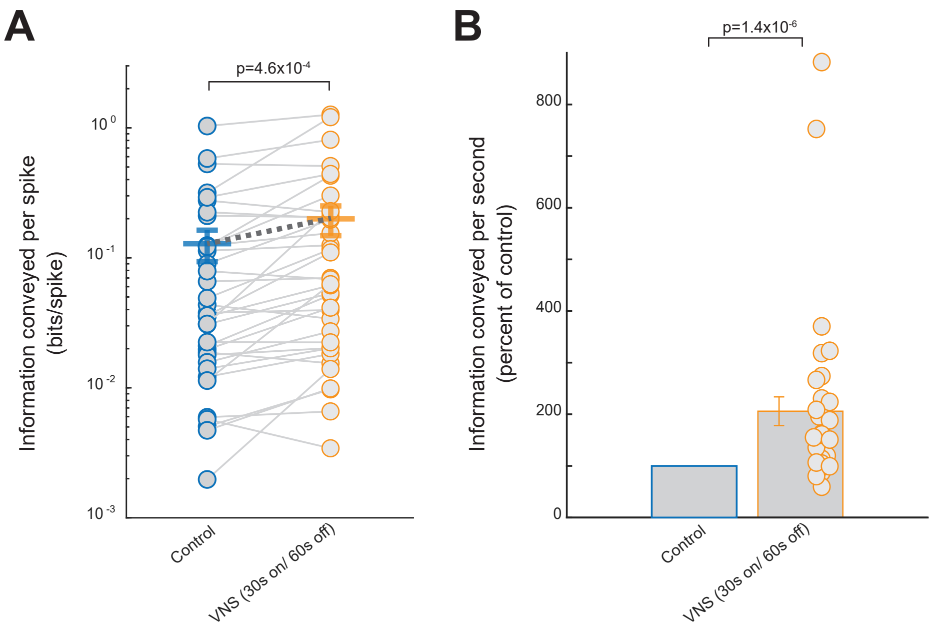

Supplementary Figure 1. Summary of improvement of information transmission efficiency (bits/spike) (A) and rate (bits/sec) (B) with standard duty-cycle VNS. Error bars indicate SEM.

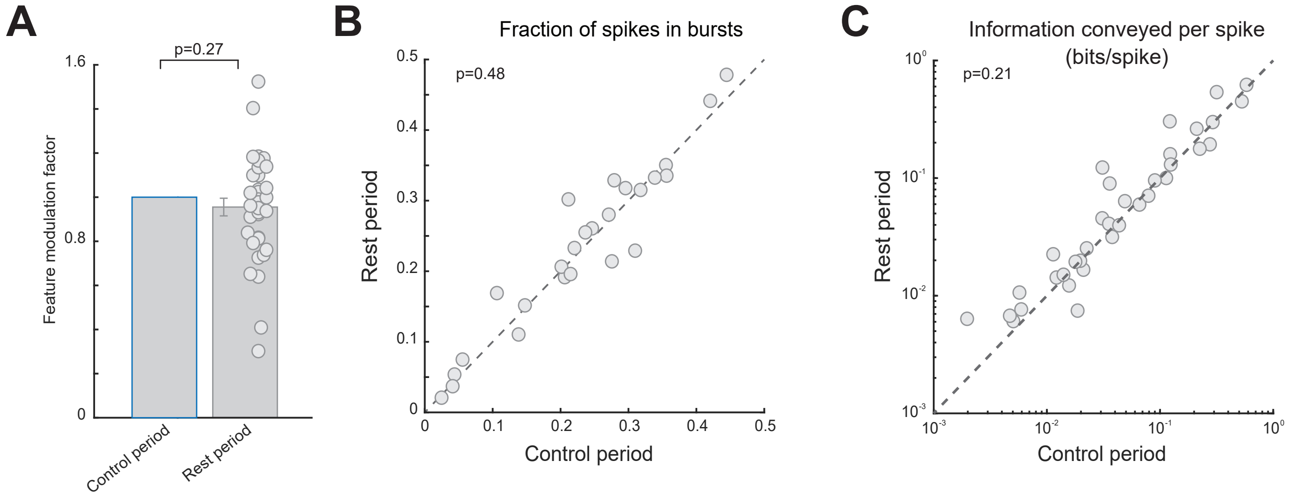

Supplementary Figure 2. VNS enhancement of sensory processing was transient with effects vanishing within 60 s of VNS cessation. A) *Summary of feature modulation factor during during the control period vs the end of the rest period.* ***B)*** *Summary of percent of spikes in bursts during the control period vs the end of the rest period.* ***C)*** *Summary of improvement in information transmission efficiency during the control period vs the end of the rest period. Error bar indicates SEM.*

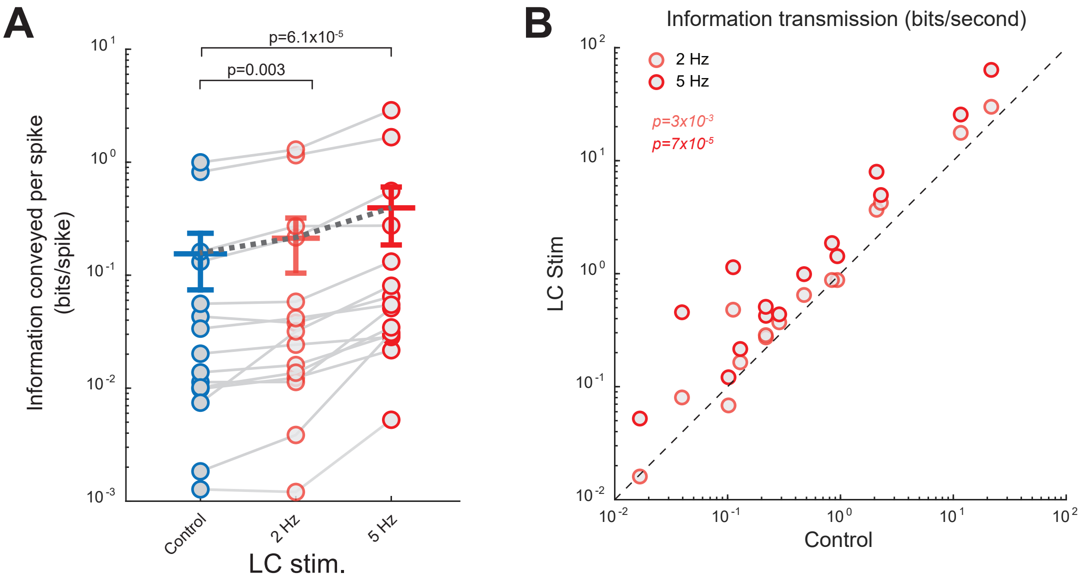

Supplementary Figure 3. Summary of information transmission efficiency (bits/spike) (A) and rate (bits/sec) (B) with and without direct LC stimulation. Error bars indicate SEM.

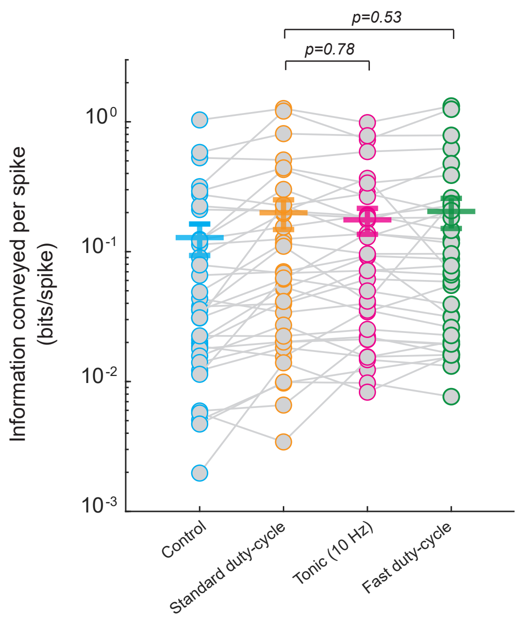

Supplementary Figure 4. Summary of information transmission efficiency (bits/spike) with different VNS patterns. Error bars indicate SEM.
